# Supplementary material for: TMcin RiPP biosynthesis in the cellular membrane
Source: bioRxiv. 2026 Jun 1:2026.05.31.729130. Preprint. [Version 1] doi: 10.64898/2026.05.31.729130 (PMC13252029; doi:10.64898/2026.05.31.729130)
Supplement: Supplement 1 [file media-1.pdf]

Supporting Information for

TMcin RiPP biosynthesis in the cellular membrane

Fauzia H. Nur[1,2], Ama N. Antwi[1,2], and Seth W. Dickey[1,2,*]

Affiliations

[1]Department of Veterinary Medicine, University of Maryland, College Park, MD, United States of America

[2]Virginia-Maryland College of Veterinary Medicine, College Park, MD, United States of America

*Correspondence to: sdickey@umd.edu

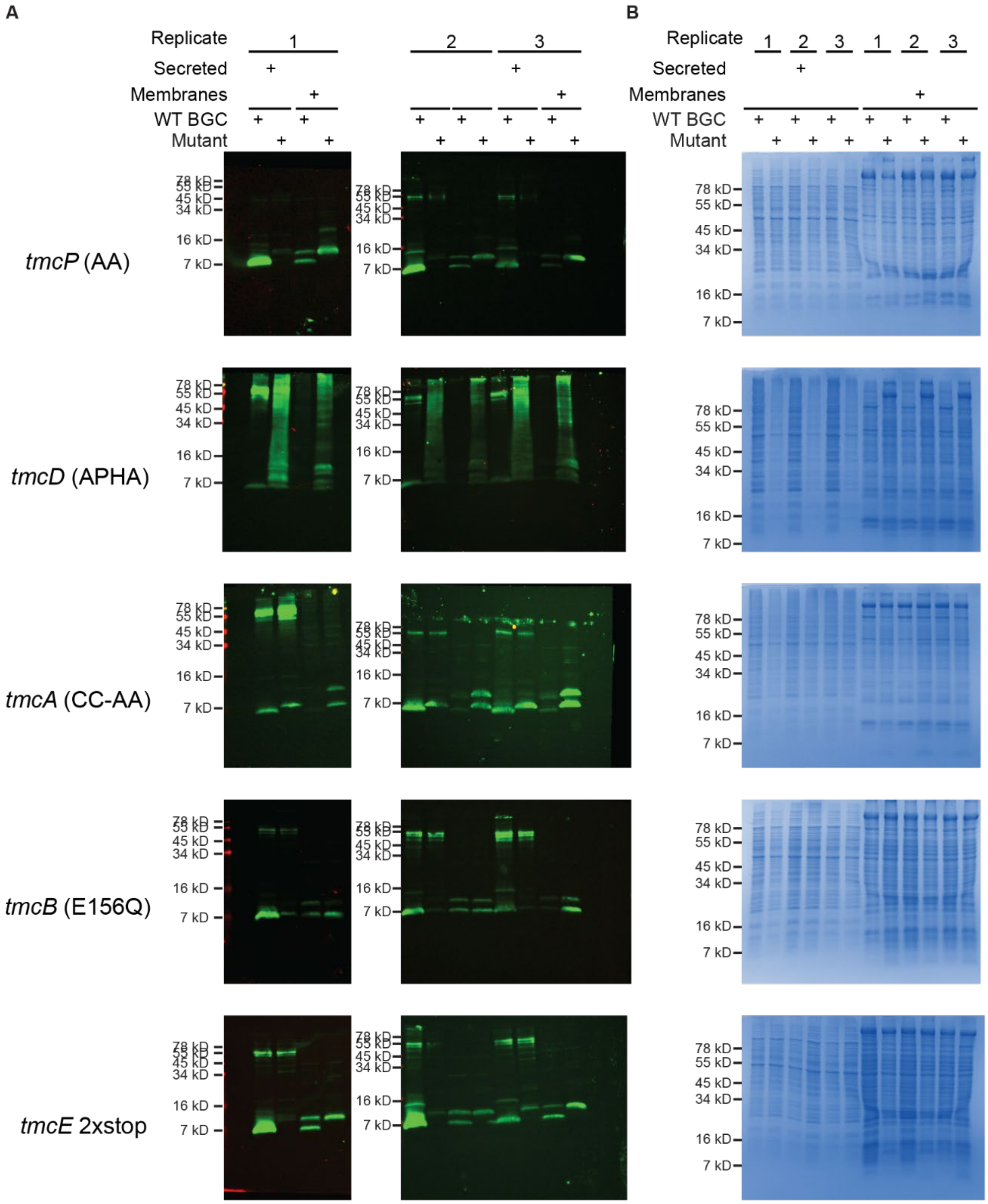

Fig. S1: Replicates of G1905 P_{tet}-TMcinBiosynthesis cells after induction of TMcin biosynthesis. (*A*) Western blots using αTMcin-G1905 and (*B*) Coomassie-stained SDS-PAGE gels of subcellular fractions after induction of TMcin biosynthesis (n = 3 biological replicates). SDS-PAGE gels stained with Coomassie blue.

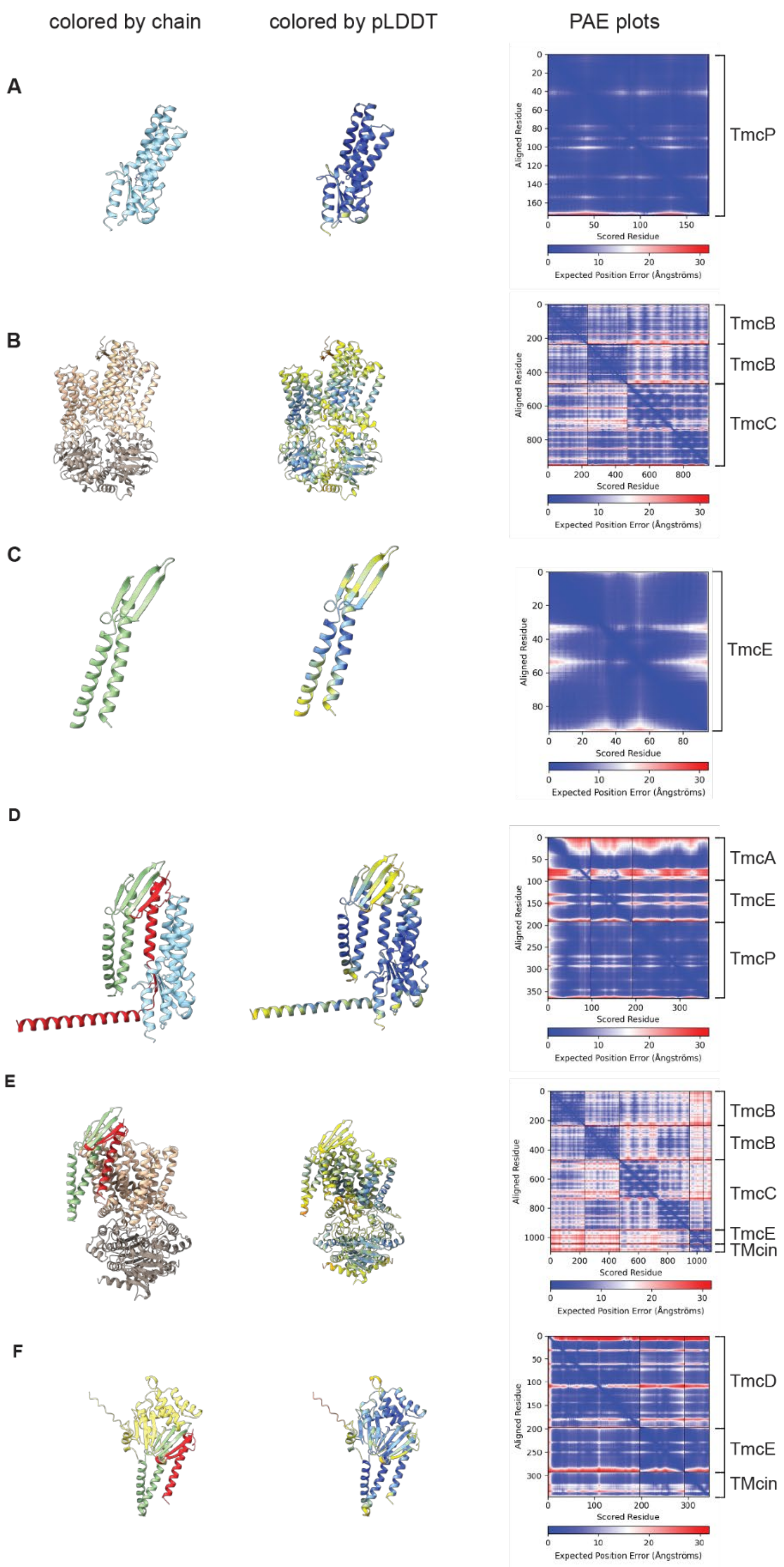

Fig. S2: **Confidence metrics and structures of AlphaFold3 generated models in this study.**

(*A-F*) Cartoon representation of proteins and complexes colored by chain (left) and pLDDT score

(middle). Predicted aligned error plots are on the right. (*A*) TmcP (one copy, sky blue). (*B*) TmcBC with two copies of TmcB (brown) and one copy of TmcC (light brown). (*C*) TmcE (one copy, sage green), (*D*) TmcE-TmcA-TmcP complex. One copy each of TmcE (light green), TmcA (red), and TmcP (cyan). (*E*) TmcE-TMcin-TmcBC complex. (*F*) TmcD-TmcE-TMcin complex. One copy each of TmcD with the predicted signal peptide removed (yellow, residues 21-217), TMcin (red), and TmcE (light green).

Table S2: Results from HMMScan using the TmcP sequence as input.

Family id	Family Accession	Clan	Env. Start	Env. End	Model Start	Model End	Bit Score	Ind. E-value	Cond. E-value	Description
DUF3267	PF11667	CL0126	61	171	2	100	26.5	8.50e-06	1.06e-09	Putative zincin peptidase

Table S3: Oligonucleotides used in this study.

Oligonucleotide name	Sequence (5’-3’)	Purpose
pCT545_antitoxin_forward	CATATGAGCACAAACCCAAA	G1905 ΔpCT545
pCT545_2Stop_antitoxin_reverse	TAATACTTAATATTTGTTTTGATAATAGCACCG	G1905 ΔpCT545
1kb_*tetML*_Forward	AATCGAATGGAAAGTCTAAAAGG	G1905 Δ*tetML*
*tetML*_1kb_Reverse	TTGCAGTTTTCCATCACG	G1905 Δ*tetML*
delta_*tetL*_Right	TAAATCGTTAAGGGATCAACTTTG	G1905 Δ*tetML*
delta-*tetM*_Left	GTGATTTTCCTCCATTCAAAAAC	G1905 Δ*tetML*
pTX15 MCS Left	GATATCCTCCTCAGCTGGATCCTACATTTTAGTTGGTTAATTT	pKX17 (introduce multiple cloning site)
pTX15 MCS Right	ATATGATTTGCGGCCGCATACGCGTGTGATGAACGCTTTATCTTAATGC	pKX17 (introduce multiple cloning site)
pKX17_delta_PxylA_ApaI_Right	TATGCTGGGCCCAGCTGAGGAGGATATCATAT	pKT17 (remove *xylR* and P_{xylA})
pKX17_delta_*xylR*_Left	GATCATCATGACAGATCCGG	pKT17 (remove *xylR* and P_{xylA})
pIMAY_*tetR*_Reverse	TGAAGTTACCATCACGGA	pKT17 (*tetR* and P_{tet})
pIMAY_Pxyl_ApaI_Forward	CTGAGGGCCCCTCTATCAATGATAGAGAGCTTATTTTAATTATACTCTATCAATGATAG	pKT17 (*tetR* and P_{tet})
*tetR*_remove_NdeI_Left	TGCGGATTAGAAAAACAACTTAAAT	pKT17 (remove NdeI site in *tetR*)
*tetR*_remove_NdeI_Right	aattATCAATTCAAGGCCGAATAAG	pKT17 (remove NdeI site in *tetR*)
*tmcB*_NdeI_Forward	TCATGTCATATGTTGAAAATTGAAAATGTATCATTTAAGTATAC	pKT17 BGC-core: insert *tmcB-tmcA*
*tmcA*_NotI_Reverse	ATACCAGGCGGCCGCTTATCTTTTGCACTCAACATCG	pKT17 BGC-core: insert *tmcB-tmcA*
pRB_Δ_GramN_Left	GCATCTGTGCGGTATTTCA	Delete Gram-negative replication and antibiotic resistance marker
pRB_Δ_GramN_Right	GTCATTACCCCAGGCGTTTA	Delete Gram-negative replication and antibiotic resistance marker
Δ_*tmcA*_Right	TAACTTTTAAACTACAAATGCGTGG	Delete *tmcB-tmcA* in pRB5gp + BGC
Δ_*tmcB*_Left	TTGTAAGTCGTCCTTTAATTTTTTTAAATATATATAATATTG	Delete *tmcB-tmcA* in pRB5gp + BGC
*tmcD*_APHA_left	TGCGTGAGGAGCTTTAAAATCTTCGAAAACTATAATTTTATTTTTTGAAC	*tmcD* APHA mutagenesis
*tmcD*_R70_Right	AGAACTCTATATAATAATATTATAAAAAAAATTGATGATGG	*tmcD* APHA mutagenesis
*tmcB*_E156Q_Forward	CAAACATTAAATGGAATGGATATTGAATCA	*tmcB* E156Q mutagenesis
*tmcB*_D155_Reverse	ATCTATTATTAGAATATTGGGTGCTATTAAC	*tmcB* E156Q mutagenesis
*tmcE*_S10L11Stop_Forward	TAATAGGTTTTTTCTATAGTAACTACCGGAAT	*tmcE* 2xstop mutation
*tmcE*_N9_Rev	GTTAATTAAATATATTAAGTGTTTCATTTCTCGAC	*tmcE* 2xstop mutation
*tmcP*_H68A_Forward	GCAGAATATATGCATATATATTTCTTGAAGAAAAAGC	*tmcP* H68A mutagenesis
*tmcP*_H68A_Reverse	TATAGCAAAAGAAAGAAGCAAACC	*tmcP* H68A mutagenesis
*tmcP*_D158A_Forward	GCAGGAGTAATGTTAATCAAAGGAATATTAaag	*tmcP* D158A mutagenesis
*tmcP*_D158A_Reverse	TCCTAATGGTGGAAGTATGTTAA	*tmcP* D158A mutagenesis
*tmcA*_C70A_Right	GCAGTAGTGAATCAACATGGTAAGTT	*tmcA* C70A mutagenesis
*tmcA*_C70A_Left	CCAAATAGTTAAACCAGCAAAAATT	*tmcA* C70A mutagenesis
*tmcA*_C95A_Left	TGCCTCAACATCGAGGCTAAC	*tmcA* C95A mutagenesis
*tmcA*_C95A_Right	AAAAGATAAGCGGCCGCA	*tmcA* C95A mutagenesis

Table S4: Plasmids used in this study.

Plasmid Name	Source
pKX15	(*35*)
pKX17	This study
pKT17	This study
pKT17+BGC-core	This study
pKT17+BGC-core *tmcB*(E156Q)	This study
pKT17+BGC-core *tmcE*(2xstop)	This study
pKT17+BGC-core *tmcP*(H68A,D158A)	This study
pKT17+BGC-core *tmcA*(C70A,C95A)	This study
pKT17+BGC-core *tmcE*(2xstop) *tmcA*(C70A,C95A)	This study
pKT17+BGC-core *tmcB*(E156Q) *tmcA*(C70A,C95A)	This study
pRB5gp	This study
pRB+BGC	(*6*)
pRB5gp+BGC	This study
pRB5gp+BGC-Δcore	This study
pRB5gp+BGC-Δcore *tmcD*(C66A,C69A)	This study
